# 1,4-Naphthoquinone Analogues: Potent Antibacterial Agents and Mode of Action Evaluation

**DOI:** 10.3390/molecules24071437

**Published:** 2019-04-11

**Authors:** Palanisamy Ravichandiran, Sunirmal Sheet, Dhanraj Premnath, Ae Rhan Kim, Dong Jin Yoo

**Affiliations:** 1 Department of Life Science, Department of Energy Storage/Conversion Engineering of Graduate School and Hydrogen and Fuel Cell Research Center, Chonbuk National University, Jeollabuk-do 54896, Korea; ravichandru55@gmail.com; 2 Department of Forest Science and Technology, College of Agriculture and Life Sciences, Chonbuk National University, 567 Baekje-daero, Deokjin-gu, Jeonju-si 561-756, Jeollabuk-do, Korea; sunirmal.micro@gmail.com; 3 Department of Biotechnology, Karunya Institute of Technology and Science, School of Agriculture and Biosciences, Karunya Nagar, Coimbatore 641114, Tamil Nadu, India; prems.bioinfo@gmail.com; 4 R&D Center for CANUTECH, Business Incubation Center, Department of Bioenvironmental Chemistry, Chonbuk National University, Jeollabuk-do 54896, Korea

**Keywords:** quinones, ROS generation, apoptosis, kinetic study

## Abstract

1,4-Naphthoquinones have antibacterial activity and are a promising new class of compound that can be used to treat bacterial infections. The goal was to improve effective antibacterial agents; therefore, we synthesized a new class of naphthoquinone hybrids, which contain phenylamino-phenylthio moieties as significant counterparts. Compound **4** was modified as a substituted aryl amide moiety, which enhanced the antibacterial activity of earlier compounds **3** and **4**. In this study, five bacterial strains *Staphylococcus aureus* (*S. aureus*), *Listeria monocytogenes* (*L. monocytogenes*), *Escherichia coli* (*E. coli*), *Pseudomonas aeruginosa* (*P. aeruginosa*) and *Klebsiella pneumoniae* (*K. pneumoniae*) were used to evaluate the antibacterial potency of synthesized naphthoquinones using the minimal inhibitory concentration (MIC) method. Most of the studied naphthoquinones demonstrated major antibacterial activity with a MIC of 15.6 µg/mL–500 µg/mL. Selected compounds (**5a**, **5f** and **5x**) were studied for the mode of action, using intracellular ROS generation, determination of apoptosis by the Annexin V-FITC/PI assay, a bactericidal kinetic study and in silico molecular modelling. Additionally, the redox potentials of the specified compounds were confirmed by cyclic voltammetry (CV).

## 1. Introduction

Bacterial infections have become the most significant threat to global human health since the Second World War, and active antibiotics have been used to cure bacterial diseases. However, the constant use of antibiotics has induced bacterial resistance, which is currently a severe problem worldwide. Therefore, the design and evaluation of active antimicrobial agents with high selectivity, less toxicity and unique novel mechanisms have become a major goal [1]. 

Apoptosis is a programmed cell death of any infected cells, which can be induced by morphological or molecular signalling mechanisms. During oral treatment of different kinds of bacterial infections, apoptosis works to kill the harmful or affected cells and maintain homeostasis in the human body. Recent studies have demonstrated that, if the chemical compound can target the infection-inducing bacterial cells, the generation of reactive oxygen species (ROS) is a significant process in apoptosis [2]. Bacterial cells are more susceptible to ROS-generating chemical agents. Hence, ROS-generating agents could provide a possible approach for advancing antibacterial drugs with high therapeutic value [3].

1,4-Naphthoquinones are widely available in many naturally occurring alkaloids and have been classified as potential antibacterial candidates [4,5]. The mechanism of these antibacterial agents involves enhanced ROS generation and is followed by apoptotic cell death [6,7]. A number of compounds with a 1,4-naphthoquinone moiety can activate noticeable biological inhibitions such as antimicrobial [8], anticancer [9], antitubercular [10], antimalarial [11] and trypanocidal [12] activities. Due to the ability to generate ROS, naphthoquinone analogues are extremely cytotoxic to the infected cells and can restrict cellular enzymes, which are responsible for apoptosis and cell growth [13]. Consequently, these compounds have been evaluated as primary models for developing and improving clinically available antibacterial drugs. However, although the mode of action for most antibacterial drugs is known, the specific mechanism of action has not yet been elucidated.

**Figure 1:**
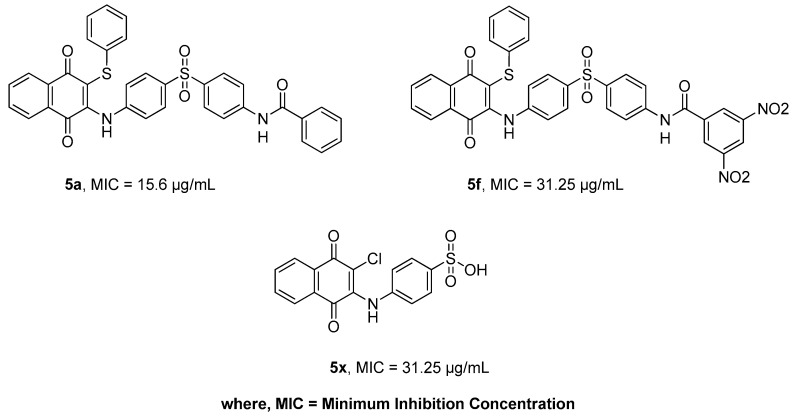
The structures of most active naphthoquinones from the current library.

We recently reported the synthesis and biological evaluation of new naphthoquinone derivatives as potential anticancer and antimicrobial agents [14,15]. The molecules were previously studied for anticancer activity, and an apoptotic mechanism in cancer cells was reported [16]. In this study, we evaluated the in vitro antibacterial effects of 1,4-naphthoquinone derivatives that contained phenylamino-phenylthio moieties as significant counterparts. We further investigated the effects of most active naphthoquinones (**5a**, **5f** and **5x**, Figure 1) for ROS generation and apoptosis induction in *Escherichia coli* (*E. coli*). In addition, a bactericidal time-kill kinetic study, in silico molecular docking and redox property by cyclic voltammetry (CV) study were also analyzed.

## 2. Results and Discussion

### 2.1. Chemistry

For synthesis, experimental details and complete results and discussion of compounds **3**–**5aa**, kindly refer to our previous report [16]. 

### 2.2. Biology

#### 2.2.1. In Vitro Antibacterial Activity

**Figure 2:**
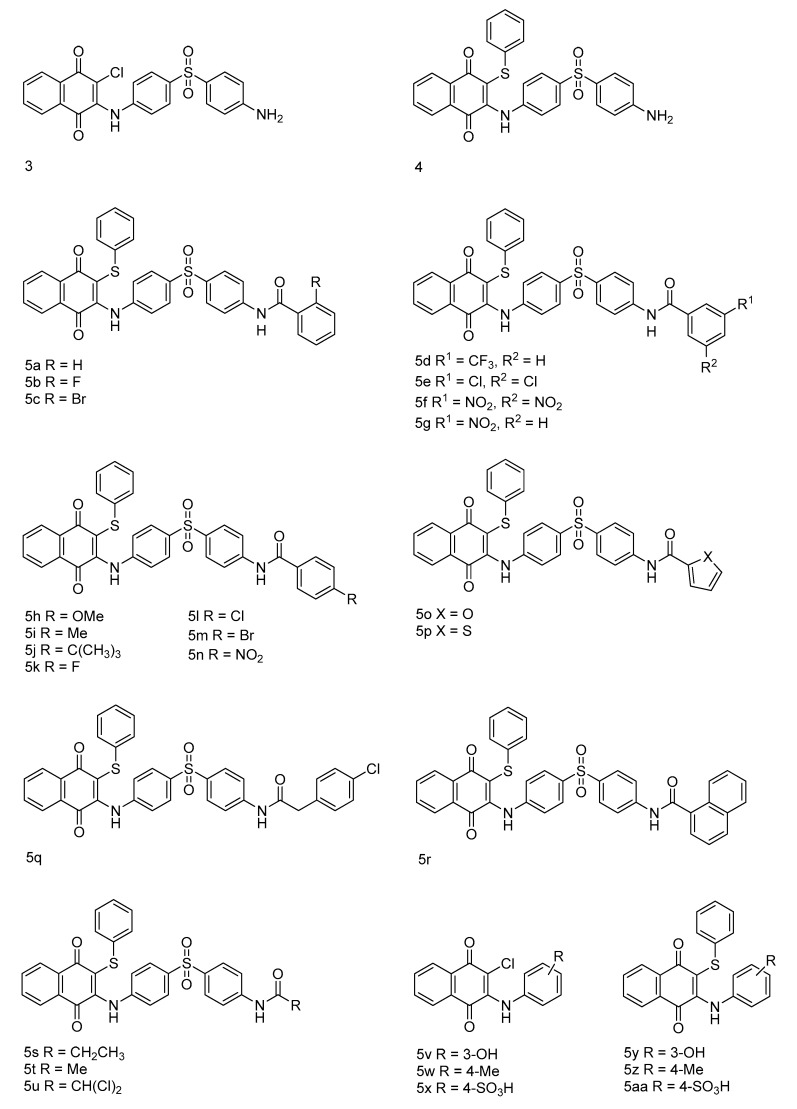
Structure of naphthoquinones (**3**–**5aa**) used in the present study.

Compounds **3**–**5aa** (Figure 2) were studied for in vitro antibacterial activity in two Gram-positive bacteria (*Staphylococcus aureus* (*S. aureus*) and *Listeria monocytogenes* (*L. monocytogenes*)) and three Gram-negative bacteria (*Escherichia coli* (*E. coli*), *Pseudomonas aeruginosa* (*P. aeruginosa*) and *Klebsiella pneumoniae* (*K. pneumoniae*)). Streptomycin was used as a positive antibacterial drug control, and MIC values are given in Table 1. 

Most of the synthesized naphthoquinone derivatives (**3**–**5aa**) showed moderate to good antibacterial activity against *S. aureus* and *E. coli*. However, these naphthoquinones had less activity toward bacterial pathogens *L. monocytogenes*, *P. aeruginosa* and *K. pneumoniae*. 

At first, compounds **3** and **4** were prepared as model compounds to evaluate antibacterial activity in Gram-positive and Gram-negative bacteria. The studied compounds showed a 64-fold weaker antibacterial potency compared to streptomycin (1.9–15.6 μg/mL). Next, varieties of substituted aromatic and aliphatic acid chlorides were introduced to compound **4** with the primary amine to create the substituted amide moiety; this resulted in more potent antibacterial agents compared with the parent compounds **3** and **4**. These results indicated that the formation of the amide moiety with various length of acid chlorides (**5a**, **5f**, **5i**, **5j**, **5o** and **5q**) significantly enhanced the antibacterial activity compared with the earlier compounds **3** and **4**. Most of the synthesized compounds were most active against the Gram-positive bacteria *S. aureus* (**5e**, **5f**, **5i**, **5k** and **5q**) with activity that ranged from 31.25 to 62.5 μg/mL, and Gram-negative bacteria *E. coli* (**5a**, **5f**, **5o**, **5s** and **5u**), with MIC values of 31.25–62.5 μg/mL. All studied compounds (**3**–**5u**) showed the weakest antibacterial activity against *L. monocytogenes*, *P. aeruginosa* and *K. pneumoniae.* In addition, compounds **5v**–**5aa** were also evaluated for in vitro antibacterial activity; compound **5w** showed significant activity against *L. monocytogenes* (62.5 μg/mL), and the other compounds **5x**, **5y** and **5z** showed potential activity against *E. coli* (31.25 to 62.5 μg/mL). From the present library (**3**–**5aa**), none of the compounds exhibited superior activity to the streptomycin positive control.

Based on these results, the structure–activity relationship (SAR) for the synthesized compounds (**3**–**5aa**) was investigated. The introduction of thiophenol into compound **3** showed moderate antibacterial activity compared with the starting compound **3**. Interestingly, the introduction of a substituted amide moiety into compound **4**, with substituted acid chlorides, resulted in increased antibacterial activity. Compound **5f** containing a 3,5-dinitro aryl moiety was found to have enhanced activity against all five bacterial pathogens studied. Similarly, compounds grafted with electron donating/electronegative functional groups showed better antibacterial activity (**5a**, **5e**, **5i**, **5k**, **5j**, **5q**, **5s**, **5x**, **5y** and **5z**) than the parent compounds **3** and **4**. However, compounds that were constructed with a bulky moiety or electron-withdrawing (except **5f**) functional groups showed poor inhibitory potencies (**5g**, **5n** and **5r**).

#### 2.2.2. Bactericidal Time-Kill Kinetic Study

**Figure 3:**
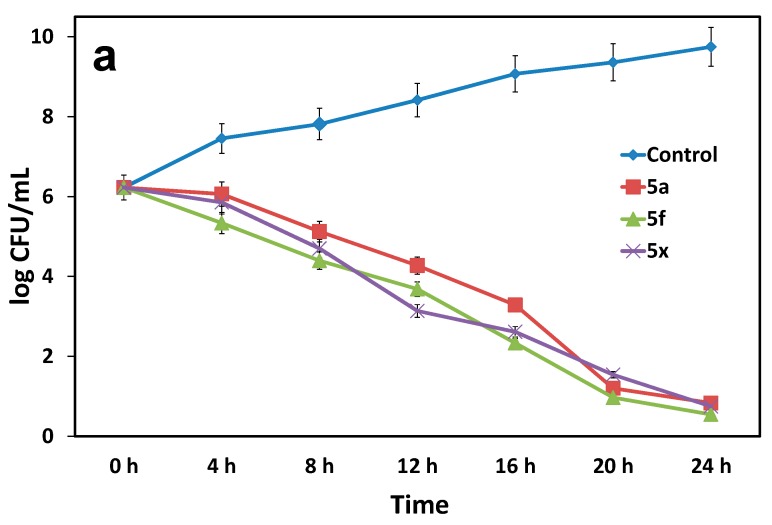

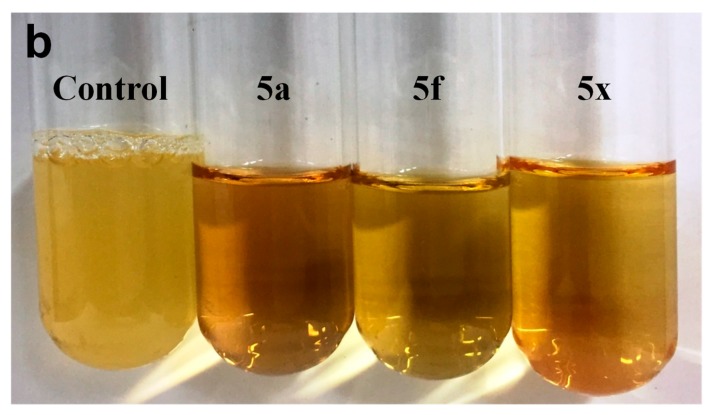
**(a**) Time-dependent bactericidal activity of compounds **5a**, **5f** and **5x** to *E. coli*. All the experiments performed in triplicates and are given the mean ± standard deviation of colony-forming units per milliliter (S.D of CFU/mL). (**b**) Images show *E. coli*, after 24 h treatment with compounds **5a**, **5f** and **5x**.

The three most active compounds **5a**, **5f** and **5x** were selected from the obtained in vitro antibacterial activity of compounds **3**–**5aa** for further investigation of the mode of action. The degree and rate of bactericidal properties must be evaluated first to determine the antibacterial potency of antimicrobial agents**.** A bactericidal time-kill kinetic study is the most convenient method to determine the degree and rate at which a compound inhibits bacteria [17]. Therefore, we first determined the bactericidal kinetic activity of **5a**, **5f** and **5x** with a time-kill assay to *E. coli*, with the corresponding MIC (15.60 µg/mL, 31.25 µg/mL and 31.25 µg/mL, respectively). *E. coli* cells at the initial stage were counted as 10^6.1^ CFU/mL, and survival started to decrease after 4 h of treatment and dropped to 10^0^ CFU/mL after 24 h. In addition, compound **5f** showed good bactericidal kinetic ability compared with **5a** and **5x**. These results clearly show time-dependent bactericidal activity in all three compounds (Figure 3).

#### Analysis of Minimum Bactericidal Concentration (MBC)

Next, the minimum bactericidal concentration (MBC) of compounds **5a**, **5f** and **5x** against *E. coli* was studied**.** The MIC and MBC are two key considerations to estimate the ratio of MBC/MIC. All the results are furnished in Table 2, the outcome of these results shows MIC ranges from 15.60 µg/mL to 31.25 µg/mL. Based on the results obtained, the characteristics of the compounds against *E. coli* can be determined. If MBC/MIC ratio is 1 or 2 the compounds are bactericidal. If MBC/MIC ratio is 4 to 16 the compounds are bacteriostatic, or else if MBC/MIC > 32, the compounds are tolerant to the bacterial strain. Therefore, the obtained results clearly indicate, the compounds **5a** and **5x** are bactericidal and compound **5f** is bacteriostatic in nature towards *E.coli* [18]. 

#### 2.2.3. Naphthoquinone-Generated ROS Detection 

**Figure 4:**
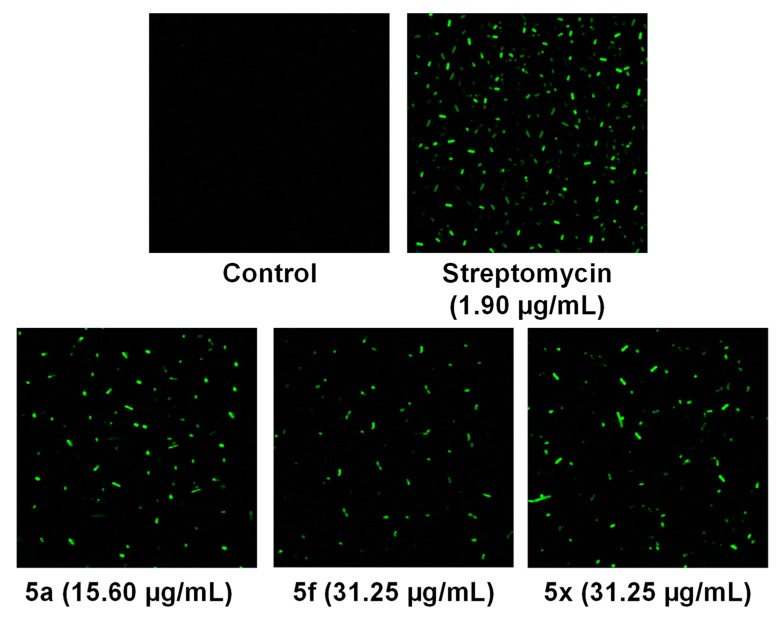
Detection of ROS generation of compounds **5a**, **5f** and **5x** in *E. coli*.

We then studied the intracellular ROS generation of the three selected compounds toward the Gram-negative bacteria *E. coli*. Enhancement of ROS level in cells is a significant requirement for the antibacterial activity of 1,4-naphthoquinones [19]. Thus, we evaluated the ROS generation of compounds **5a**, **5f** and **5x** in *E. coli* by DCFH-DA fluorescent dye staining. Treatment of *E. coli* with **5a**, **5f** and **5x** generated bursts of ROS, as shown by the increasing green fluorescence intensity due to compound-induced ROS production (Figure 4). Compounds **5a**, **5f** and **5x** showed equal fluorescence intensity to the antibacterial drug streptomycin. These results indicate that the antibacterial activity of **5a**, **5f** and **5x** impacts the oxidative stress of ROS. 

#### 2.2.4. Apoptosis by Annexin V-FITC/PI Double Staining Assay 

**Figure 5:**
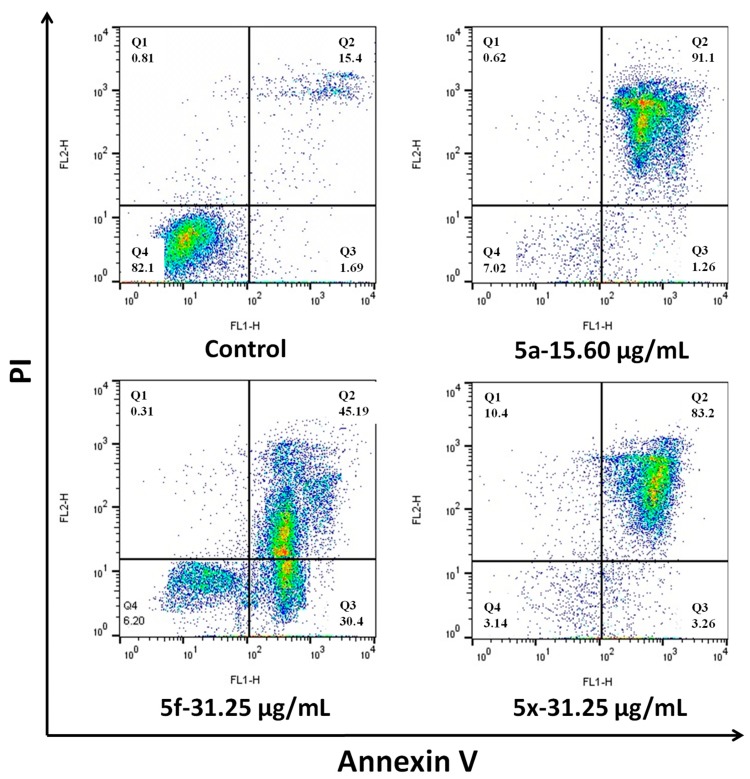
Apoptosis evaluation was performed by Annexin V-FITC/PI (Annexin V-Fluorescein isothiocyanate/Propidium Iodide (FITC/PI)) double staining assay.

Further, apoptosis-induced effects of the selected compounds **5a**, **5f** and **5x** were evaluated by the Annexin V-FITC/PI double staining assay. Bacterial cells (*E. coli*) were treated with the respective MIC of **5a**, **5f** and **5x** for 12 h; then cells were harvested, washed with PBS solution and stained with Annexin-V and PI. The total percentage of apoptotic cells was evaluated by flow cytometry. As shown in Figure 5, the treatment of *E. coli* with **5a** and **5x** at 15.60 µg/mL and 31.25 µg/mL, respectively, was identified in 91.1% and 83.2% of late apoptotic cells compared with 15.4% in control cells. However, compound **5f** showed death in 30.4% of cells in early apoptosis and 45.19% in late apoptosis. These results indicate that compounds **5a**, **5f** and **5x** induce early apoptosis and late apoptosis in *E. coli* at specified concentrations. Therefore, the apoptotic effects of compounds occur in the order **5a** > **5x** > **5f**.

#### 2.2.5. Molecular Docking Study

**Figure 6:**
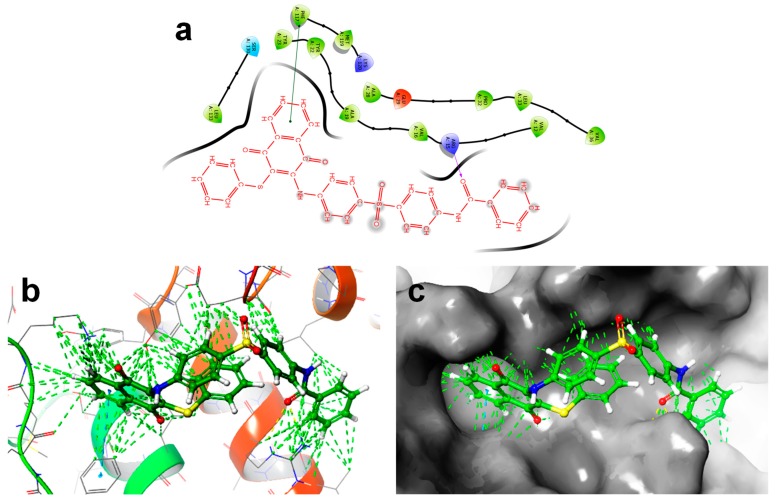
**(a**) 2D docking interaction of **5a** with the active site of 3CW0. (**b**) 3D docking of **5a** (S = −2.636 kcal/mol) in the active site of 3CW0. (**c**) Docking packing representation of **5a** with suitable binding pockets of 3CW0.

Many clinically evaluated drugs do not reach end users due to poor pharmacokinetics or intolerable side effects [20]. Consequently, in silico docking can be used to estimate the distribution, metabolism, absorption, toxicity and excretion, which may be a significant approach that can reduce these risks. In proteins, DmsD is a type of cytoplasmic chaperone that plays a significant role in blocking redox proteins from early transport. Therefore, the molecular interaction of compound **5a** was studied against the crystal structure of the *E. coli* DmsD protein (PDB ID: 3CW0), and the results are depicted in Figure 6.

A number of clinically evaluated drugs could not reach end users due to the unfortunate pharmacokinetics or intolerable side effects. Consequently, in silico docking approaches to estimate the distribution, metabolism, absorption, toxicity and excretion may be a significant approach to reduce these risks. In proteins, DmsD is a kind of cytoplasmic chaperones that play a significant role in stopping redox proteins from early transport. Therefore, the molecular interaction of compound **5a** was studied against the crystal structure of *E. coli* DmsD protein (PDB ID: 3CW0) and the obtained results are depicted in Figure 6.

The in silico molecular docking results of **5a** indicated that the molecular interactions with the biological model showed an active site of the 3CW0 protein with a docking score of -2.636 kcal/mol. The results indicate that a ligand was bound in the cavity of the protein molecule. The superposition of **5a** and amino acid residues of the 3CW0 protein are evident and depicted in Figure 6b,c. Compound **5a** was exposed to a hydrogen-bonding interaction with the corresponding active site of ARG A15 along with π–π stacking. These relationships of 3CW0 and **5a** might explain the experimental antibacterial activity; however, additional investigations are warranted to further elucidate the mode of action.

#### 2.2.6. Electrochemical Study

**Figure 7:**
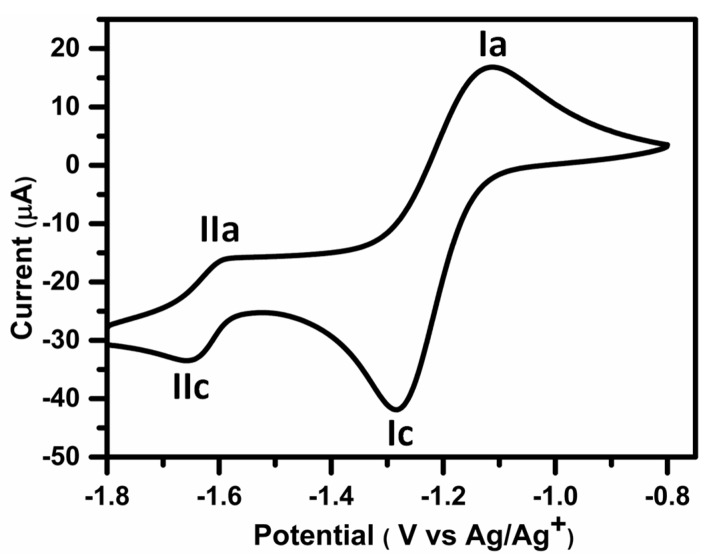
Cyclic voltammetry of **5a** (1 mM) in CH_3_CN + Bu_4_NBF_4_ (0.1 M) on glassy carbon electrode (GCE) with cathodic direction, potential range: −0.8 V up to −2.0 V, *v* = 0.1 V s^−1^.

An electrochemical study is a convenient tool for characterizing the redox-active molecules [21]. First, CV for the selected naphthoquinones **5a**, **5f** and **5x** was performed in acetonitrile (CH_3_CN + Bu_4_NBF_4_, 0.1 mol L^−1^). Cathodic and anodic peak currents were measured at a scan rate of 100 mV s^−1^ with reference to the Ag/Ag^+^ electrode (non-aqueous), and the reduction-oxidation potential (E_redox)_ values, (E_pc_ + E_pa_)/2 were determined using the midpoint of the peak potentials. The CV of **5a** is presented in Figure 7 and comprises two reduction and oxidation peaks. Similar redox peaks were observed for compounds **5f** and **5x** (Figures S1 and S2). The electrochemical measurements for each compound are shown in Table 3. The reduction order was established by comparing the first reduction potentials (Table 3, Column 6). Based on the redox potentials, the reduction potentials of the compounds are in the following order: **5a** > **5f** > **5x**. Hence, CV analysis provided insight into the valuable relationship between MIC value and E_redox_. The compounds with less negative E_redox_ values represented more active antibacterial agents. Here, **5a** showed less negative potential and exhibited a better MIC value. The obtained antibacterial activity and redox potentials of **5a**, **5f** and **5x** were correlated with their order of activity**.** Therefore, these results clearly suggest that redox behaviour is a significant factor in determining the antibacterial activity of naphthoquinones [22].

## 3. Experimental 

### 3.1. Chemistry

The chemicals, synthetic methods and instruments used for this investigation were the same as those in an earlier study. Please refer to our previous report [16] for the structural characterization of the synthesized naphthoquinones (**3**–**5aa**).

### 3.2. In Vitro Antibacterial Activity

Antibacterial activity of the tested samples (**3**–**5aa**) was determined by the MIC method. The standard strains were obtained from American type culture collection (ATCC), Korean collection type culture (KCTC) and Korean Agricultural Culture Collection (KACC). In detail, pre-activated (sub-cultured in fresh Luria–Bertani Broth medium (BD Difco™, Houston, TX, USA) at 37 °C under shaking conditions (220 rpm) in an incubator overnight) *S. aureus* (ATCC 23235), *L. monocytogenes* (ATCC 19111), *E. coli* (KCTC 2571), *P. aeruginosa* (KACC 10259) and *K. pneumoniae* (ATCC BAA-2342) were inoculated separately in 5 mL of LB liquid medium and incubated at 37 °C until the log growth phase was achieved (optical density, OD_600_ value ~ 0.6). After incubation, the bacterial cell culture was centrifuged (8000 rpm for 10 min.) and washed with a phosphate-buffered solution (PBS). The cell pellets were suspended in PBS solution to attain a final density of 10^6^ cells/mL (standardized spectrophotometrically OD_540_ ~ 1.0). Bacterial suspensions in Luria–Bertani Broth medium at initial inoculums of 10^6^ cells/mL were added to polystyrene 96–well plates and exposed to the synthesized naphthoquinones (**3**–**5aa**) at sufficient concentrations (range: 0.05 µg/mL to 1000 µg/mL) for 24 h at 37 °C. Dimethyl sulfoxide (DMSO) was used as a solvent. The MIC was the lowest drug concentration at which observable bacterial growth was inhibited. Commercially available streptomycin was used as a reference compound. All tests were performed in triplicate [23].

### 3.3. Bactericidal Time-Kill Kinetic Study

*E. coli* were seeded for 12 h and diluted to 1:10000 in Mueller Hinton Broth (MHB) medium. The cells were cultured at 37 °C with aeration of 225 rpm for 2 h. Compounds **5a**, **5f** and **5x** were treated with *E. coli* at respective MIC values in culture tubes at 37 °C at 225 rpm. At regular pauses, 100 µL of bacterial solution was placed in a 96–well plate, centrifuged for 3 min and resuspended in 100 µL of 1 × PBS. Serially diluted bacterial suspensions were placed and nursed on Mueller Hinton Agar (MHA) plates at 37 °C overnight. Finally, any colonies that formed were counted, and CFU per mL was calculated. All experiments were performed in triplicate [24]. 

#### Analysis of Minimum Bactericidal Concentration (MBC)

By subculture method, MBC was determined. At first, the concentration of samples **5a, 5f** and **5x** were prepared at their respective MIC concentrations. Next, the samples inoculated and incubated onto agar for 24 h in Petri plates at 37 °C. MBC was calculated as 99.9 % of the early bacterial inoculum was killed by the lowermost concentration of samples (< 0.01 % of survivors). All experiments were carried out in triplicate [25]. 

### 3.4. Naphthoquinone-Generated ROS Detection

2′,7′-Dichlorofluorescein-diacetate (DCFH-DA), a fluorescent dye, was used to measure the generation of ROS [26]. *E. coli* was incubated, washed with PBS and resuspended in PBS buffer (pH-7.2). Bacterial cells were stained with 10 μM DCFH-DA for 30 min at 37 °C in the dark. The cells were then washed with double distilled water to remove the unreacted DCFH-DA. Next, DCFH-DA-treated bacterial cells were incubated with compounds **5a**, **5f** and **5x** (15.6 µg/mL, 31.25 µg/mL and 31.25 µg/mL, respectively) for 4 h. Finally, the ROS generation of samples incubated with *E. coli* was determined by fluorescence microscopy (LSM 510 META-Carl Zeiss, Jena, Germany) installed at the Center for University-Wide Research Facilities (CURF) at Chonbuk National University (CBNU, Jeollabuk-do, Republic of Korea). The excitation–emission wavelengths were fixed at 488 nm and 535 nm, respectively [27]. Streptomycin was used as a positive control (1.90 µg/mL). 

### 3.5. Apoptosis Determination by Annexin V-FITC/PI Assay

After treatment with compounds **5a**, **5f** and **5x**, the infected *E. coli* cells were assessed with the FITC-Annexin V/PI using a BD FACSCalibur flow cytometer (San Jose, California, USA). Compounds with corresponding MICs were treated with bacterial solution (1 × 10^7^) for 12 h. After incubation, to remove any excess of bacterial culture, the bacterial solution was centrifuged and washed with PBS (pH 7.2). The accumulated cell pellet was resuspended in Annexin V binding buffer. The staining protocol was performed according to the manufacturer’s instructions. Finally, the stained cells were counted using a BD FACSCalibur flow cytometer (San Jose, California, USA). The acquired data were isolated using FlowJo 10.0.7 software (Treestar Inc, Ashland, OR, US) [28].

### 3.6. In Silico Molecular Docking Study

An in silico docking study was performed to understand the binding mechanism of **5a**, using the crystal structure of *E. coli* DmsD (PDB ID: 3CW0) [29]. Prior to molecular docking, the crystal structure of the *E. coli* receptor was prepared using the protein preparation wizard in Schrödinger Maestro (version 8.5, Schrödinger, LLC, New York, USA). The crystal structure was transferred from the protein data bank (PDB), and the chemical structures of the compounds were drawn using ChemDraw Professional 15.1. The docking study was performed using an Extra Precision (XP) mode docking protocol. The XP GS score was calculated to evaluate the docking output, and PyMOL was used to determine the binding mode [30]. 

### 3.7. Electrochemical Study

The electrochemical experiment was performed on a potentiostat/galvanostat/ZRA Gamry model REF600 device with a three-electrode system. Glassy carbon, platinum (Pt) wire and Ag/Agcl (non-aqueous) electrodes were employed as working, counter and reference electrodes, respectively. Tetrabutylammonium tetrafluoroborate (0.1 M) in CH_3_CN was used as a supporting electrolyte. CV was run at 100 mV s^−1^, and scanning of the window potential started toward the positive and then the negative directions. Compounds **5a**, **5f** and **5x** were dissolved in DMSO at fixed concentrations of 1 mM and were added to the supporting electrolyte (12 mL). The CV measurement was carried out in a nitrogen environment [31].

## 4. Conclusions

In summary, this study effectively demonstrated successful synthesis of naphthoquinone derivatives and in vitro antimicrobial evaluation of the compounds. A mechanism of action for naphthoquinone derivatives to *E. coli* that involves bacterial membrane binding was confirmed by intracellular ROS generation, apoptosis induced effects and a bactericidal time-killing study. In addition, molecular modelling and an electrochemical study were established to support the mechanism of antibacterial activity. Thus, the obtained results indicate that additional studies are needed to determine the clinical applications of these synthesized compounds as potent antibacterial candidates for *E. coli*. These compounds could be applied in the future to target and control *E. coli* infections, which is one of the most predominant microorganisms in hospitalized infections.

## Figures and Tables

**Table 1:** In vitro antibacterial activity of 1,4-naphthoquinones (**3**–**5aa**) expressed as MIC (μg/mL).

MIC (μg/mL)					
	Gram-positive		Gram-negative		
Compd.	*S. aureus* (ATCC 23235)	*L. monocytogenes* (ATCC 19111)	*E. coli* (KCTC 2571)	*P. aeruginosa* (KACC 10259)	*K. pneumoniae* (ATCC BAA-2342)
3	500	250	NA	500	250
4	125	500	250	250	125
5a	125	125	**15.6**	125	250
5b	500	500	500	500	500
5c	500	250	250	NA	500
5d	125	500	500	500	500
5e	**62.5**	500	250	500	250
5f	**31.25**	125	**31.25**	**62.5**	**62.5**
5g	500	250	500	250	250
5h	125	500	250	250	500
5i	**62.5**	500	500	250	500
5j	250	125	250	**62.5**	250
5k	**62.5**	500	500	500	500
5l	125	500	125	125	500
5m	250	NA	250	125	250
5n	125	500	250	500	NA
5o	500	NA	**62.5**	250	125
5p	250	NA	NA	NA	250
5q	**62.5**	500	250	500	250
5r	250	500	250	125	250
5s	500	500	**62.5**	500	500
5t	500	250	NA	500	500
5u	500	NA	**62.5**	250	250
5v	500	250	NA	500	500
5w	500	**62.5**	NA	500	250
5x	125	250	**31.25**	500	125
5y	250	NA	**62.5**	250	125
5z	250	500	**62.5**	250	125
5aa	250	500	500	250	250
Str ^[*]^	7.70	15.61	1.90	7.81	7.83

Entries in boldface highlight MIC values lower than 100 μg/mL; NA-No activity; ^[*]^ Streptomycin.

**Table 2:** MBC and MBC/MIC data of compounds **5a, 5f** and **5x** against *E. coli.*

Compound	MIC (µg/mL)	MBC (µg/mL)	MBC/MIC Ratio
**5a**	15.60	31.2	2.0
**5f**	31.25	93.75	3.0
**5x**	31.25	62.5	2.0

**Table 3:** The major electrochemical parameters of selected naphthoquinones (c = 1 mmol/L), using CV on GCE, in CH_3_CN/Bu_4_NBF_4_, 0.1 mol/L, *v* = 100 mV s^−1.^

Compound	Epa_1_ (V)	Epa_2_ (V)	Epc_1_ (V)	Epc_2_ (V)	E_redox_ (V)
**5a**	−1.111	−1.598	−1.279	−1.647	−1.194
**5f**	−1.137	−1.545	−1.254	−1.642	−1.195
**5x**	−1.413	−1.533	−1.283	−1.614	−1.348

## Supplementary Materials

The following are available online at https://www.mdpi.com/1420-3049/24/7/1437/s1. Figure S1 and Figure S2: Electrochemical study of compounds **5f** and **5x**, respectively.
