# Supplementary material for: 1,4-Naphthoquinone Analogues: Potent Antibacterial Agents and Mode of Action Evaluation
Source: Molecules. 2019 Apr 11;24(7):1437. doi: 10.3390/molecules24071437 (PMC6480589; doi:10.3390/molecules24071437)
Supplement: Supplementary file 1 [file molecules-24-01437-s001.pdf]

molecules

Supplementary Material

1,4-Naphthoquinone Analogues: Potent Antibacterial Agents and Mode of Action Evaluation

Palanisamy Ravichandiran [1], Sunirmal Sheet [2], Dhanraj Premnath [3], Ae Rhan Kim [4,*] and Dong Jin Yoo [1,*]

1 Department of Life Science, Department of Energy Storage/Conversion Engineering of Graduate School, and Hydrogen and Fuel Cell Research Center, Chonbuk National University, Jeollabuk-do 54896, Republic of Korea; ravichandru55@gmail.com
2 Department of Forest Science and Technology, College of Agriculture and Life Sciences, Chonbuk National University, 567 Baekje-daero, Deokjin-gu, Jeonju-si 561-756, Jeollabuk-do, Republic of Korea; sunirmal.micro@gmail.com
3 Department of Biotechnology, Karunya Institute of Technology and Science, School of Agriculture and Biosciences, Karunya Nagar, Coimbatore-641114, Tamil Nadu, India; prems.bioinfo@gmail.com
4 R&D Center for CANUTECH, Business Incubation Center, Department of Bioenvironmental Chemistry, Chonbuk National University, Jeollabuk-do 54896, Republic of Korea
* Correspondence: djyoo@jbnu.ac.kr (D.J.Y.); canutech@hanmail.net (A.R.K.); Tel.: +82-(0)63-270-3608/+82-(0)10-2110-2328 Fax: +82-(0)63-270-3909 (D. J. Y.)

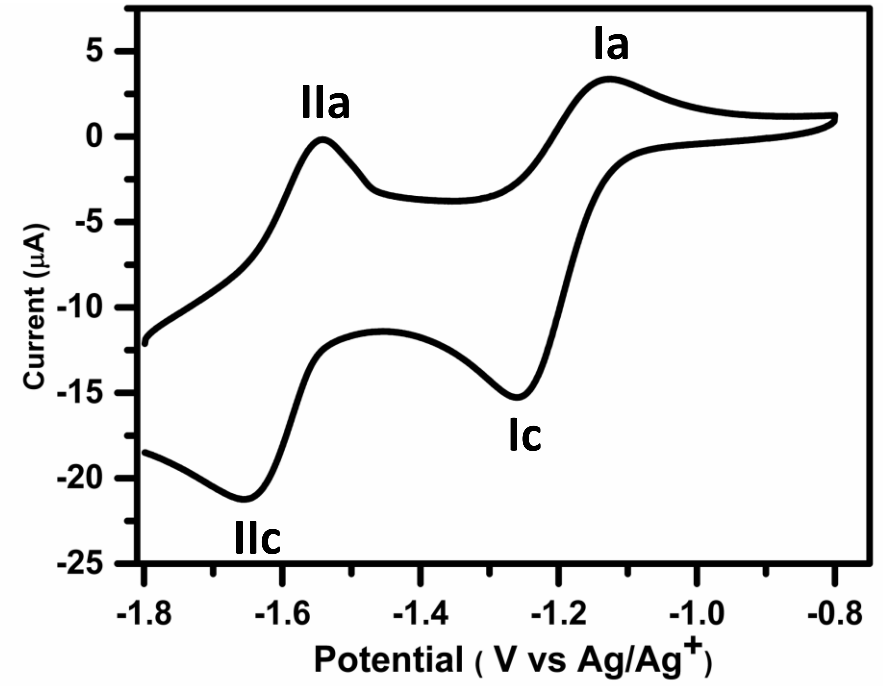

Figure 1. Cyclic voltammetry (CV) of **5f** (1 mM) in CH_3CN + Bu_4NBF_4 (0.1 M) on glassy carbon electrode (GCE) with cathodic direction, potential range: -0.8 V up to -2.0 V, v = 0.1 V s^{-1}.

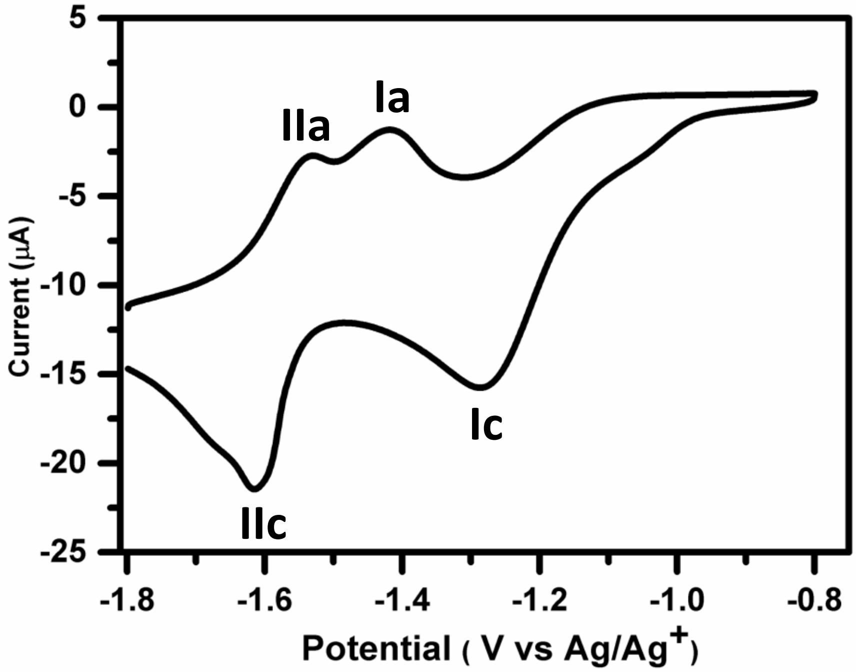

Figure S2. Cyclic voltammetry (CV) of **5x** (1 mM) in CH_3CN + Bu_4NBF_4 (0.1 M) on glassy carbon electrode (GCE) with cathodic direction, potential range: -0.8 V up to -2.0 V, v = 0.1 V s^{-1}.
